# A review of active learning approaches to experimental design for uncovering biological networks

**DOI:** 10.1371/journal.pcbi.1005466

**Published:** 2017-06-01

**Authors:** Yuriy Sverchkov, Mark Craven

**Affiliations:** Department of Biostatistics and Medical Informatics, University of Wisconsin–Madison, Madison, Wisconsin, United States of America; University of California, Berkeley, UNITED STATES

## Abstract

Various types of biological knowledge describe networks of interactions among elementary entities. For example, transcriptional regulatory networks consist of interactions among proteins and genes. Current knowledge about the exact structure of such networks is highly incomplete, and laboratory experiments that manipulate the entities involved are conducted to test hypotheses about these networks. In recent years, various automated approaches to experiment selection have been proposed. Many of these approaches can be characterized as active machine learning algorithms. Active learning is an iterative process in which a model is learned from data, hypotheses are generated from the model to propose informative experiments, and the experiments yield new data that is used to update the model. This review describes the various models, experiment selection strategies, validation techniques, and successful applications described in the literature; highlights common themes and notable distinctions among methods; and identifies likely directions of future research and open problems in the area.

## Introduction

Biological processes are complex, involving many interactions among elementary units. To describe such processes, a common representation is a network model. For example, gene regulatory networks (GRNs) describe activation and inhibition relationships among genes, metabolic networks describe which enzymes yield which metabolites in a metabolic pathway, etc. Such biological networks are often quite large, with GRNs containing thousands of genes, for example. Current knowledge of many such networks is largely incomplete, and the primary means of uncovering details of these networks is through laboratory experiments.

In order to design an experiment that effectively adds to current knowledge, it is necessary to understand the current state of knowledge, as well as to have an idea of what the outcome of the experiment will add. What outcomes are likely? What would one conclude from each outcome? When the biological network in question is complex, and when there is uncertainty about the details of the network, these questions become hard to answer. Furthermore, the number of potential experiments from which to choose is often quite large. For example, when considering single gene knockdown experiments, there are as many potential experiments as there are genes, and when considering double knockdowns, the number of possibilities is quadratic in the number of genes. It is this challenge that creates a need for computational solutions to experimental design. This review discusses various methods that address this problem by using algorithms for the selection of experiments.

**Fig 1:**
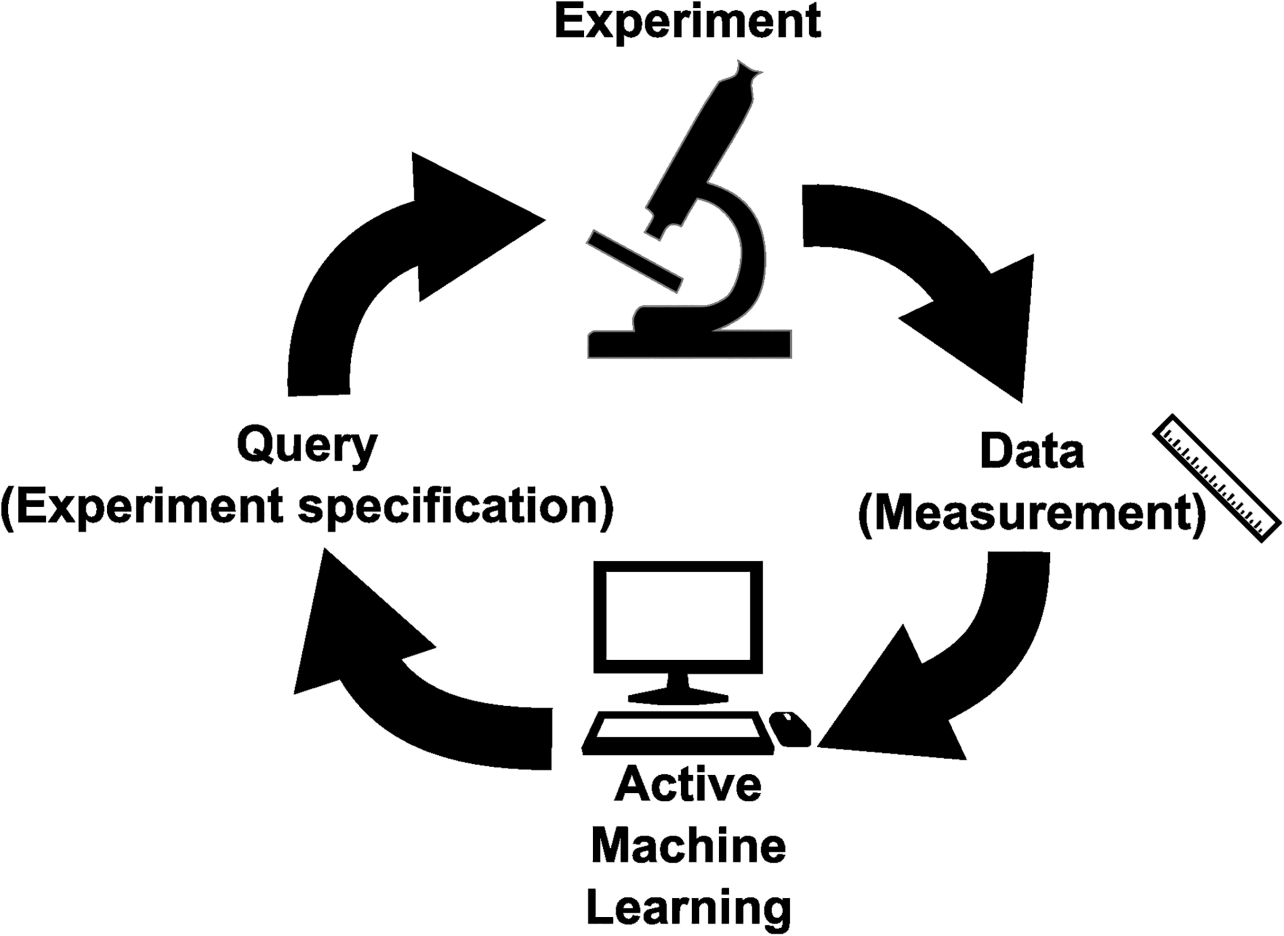
The active learning loop. In active machine learning, data from experiments informs a learner that formulates queries for further experiments that are expected to be most informative for refining a model.

Active learning approaches for uncovering biological networks are meant to solve the problem of designing experiments when human decision-making is less than optimal for the task. Active machine learning is a subfield of machine learning that studies algorithms that select the data they need for the improvement of their own models. In a standard machine learning setting, an algorithm learns some sort of model from a sample of data, and the model is subsequently used for one of a variety of purposes (classification, clustering, prediction, etc.). This can be viewed as a linear pipeline from data to some end application of that data. Active machine learning adds an iterative refinement process to the pipeline. In addition to the machine learning component that learns a model from data, there is a component that uses the model to characterize what sort of additional data would best improve the model further. We call this characterization a query. In the context of experimental design, the query specifies the manipulation to be performed in the experiment, such as which gene(s) to knock down in a knockdown experiment or what growth media to use in a growth experiment. The active learner selects a query that would be most informative for improving the model. In response to the query, new data, such as measurements performed under the conditions specified by the query, are used to update the model accordingly. This cycle can be repeated multiple times until the model is sufficiently accurate. This process forms the active learning loop (Fig 1).

In the particular setting of uncovering biological networks, the data is often gene expression data, but it can also be some other measurement of abundances of biological molecules involved in the network of interest or measurements of a reporter variable. The modeling component typically involves a mathematical representation of the network structure, often mirroring the believed causal relationships among the entities involved. Typically, the network inference process yields a collection of possible models that explain prior data equally well (or a distribution of models in which there are multiple likely models), and the challenge of improving the model is that of narrowing down this collection through additional experiments. The details of how this is accomplished depend on the modeling component (specifically, what the mathematical model is and how it is learned from data) and on some criterion or strategy to select experiments based on this model.

This review discusses a variety of works that fall in this category of approaches. The unique contributions of each work are highlighted throughout. Additionally, there are aspects that are present in all methods. First, there is the modeling component, which is essential in every machine learning and active machine learning method. There are many approaches to modeling biological networks in general. The methods we discuss are focused on either inferring a mathematical representation of the biological network from data or constructing it based on a combination of data and prior knowledge. The mathematical models we cover include models that represent variables as having discrete states (e.g., a gene being active or inactive) and models that represent variables as continuous quantities (e.g., protein abundances). Among those models, there are models with deterministic dynamics, wherein the value of one variable is a function of another, and models with stochastic dynamics, wherein the value of one variable is a random quantity sampled from a distribution that depends on the value of another variable. The biological networks covered by these methods include metabolic, signaling, and regulatory networks, as well as networks that describe combinations of these three groups of interactions.

Second, there are the queries that specify the experiments and the various types of responses to the queries. The types of possible queries correspond to various perturbations of the biological network that can be carried out in an experiment. These include changing the activity of specific genes, manipulating the abundances of specific proteins, changing the environment by specifying growth media, or applying some combination of these perturbations. The response to a query—the data collected in an experiment—also varies: it may be a multidimensional measurement that informs of the states of all or many of the entities in the model, such as a gene expression profile, or it may be a one-dimensional measurement, such as cell growth.

Third, there are the criteria for selecting experiments. The essential component that characterizes active machine learning is the criterion by which additional data (experiments) are selected to improve the model. Although the details of the experiment selection criterion are dependent on the particular model representation, there are some general themes that undergird all criteria. Ideally, the criterion selects experiments in such a way that the model can be most improved at the least cost. Each method discussed here uses a criterion based on either entropy, a difference between experimental outcomes, or an expected cost. The generalization of these common principles can direct future work, such as the application of active learning to new models.

Fourth, there is the approach to evaluating the methods. Ideally, to evaluate an active learning approach, one would run a sequence of active-learning-selected experiments and measure the cost (or number of experiments taken) to arrive at a sufficiently accurate model. However, it is typically impractical to do this for the purpose of merely testing the active learning strategy. Additionally, the true model of the biological system is unknown (hence the need for the method). This leads to two common approaches for evaluating active learning methods. One approach is to use some generative model to simulate a system that is like the biological system of interest and use these simulated experiments as input to the active learning method. An advantage of this approach is that the ground truth model is known, and it is easy to check the extent to which the model learned by the active learning algorithm matches the true generating model. Another approach is to use data from real, previously performed experiments. In this setup, data from an experiment is held out until the active learning algorithm proposes to perform that experiment, and the model performance is measured by how accurately it predicts the results of experiments it has not observed or how close the model is to one learned from all available data. In the case of biological networks, models learned from real data can also be validated by checking whether the information they represent matches previously discovered knowledge about the organisms studied. All of these approaches test the method without expending resources to actually perform new experiments.

Finally, a few of the methods discussed here have been applied in practice and validated against the literature or in lab experiments in terms of biological findings. In two cases, experiments were performed according to the algorithms' recommendations, and the experimental results led to gains in knowledge about the systems studied.

### Mathematical notation

In mathematical formulae, we use lowercase, nonbold type for the atomic elements of sets, scalar values, and values taken by random variables; uppercase type for sets, random variables, and matrices; and bold type for vectors and matrices. We use ℙ to represent probabilities, *f* to represent probability density functions, and E to represent expectations. The mathematical notation that refers to entities that appear in most methods is kept as consistent as possible throughout this review: *m* ∈ *M* represent models (or their closest equivalents); observed states are represented by *x* or its bold and uppercase variants, depending on whether states are seen as atomic set elements, vectors, or random variables within the context of a particular method; and similarly, *e* or its bold and uppercase variants represent experiments.

The structure of the remainder of this review is as follows. The reviewed works are divided into two categories that correspond the following two sections, "Active learning de novo" and "Active learning with prior knowledge." We discuss the previously enumerated aspects for the approaches within these sections, and following them, a discussion concludes the review.

## Active learning de novo

This section summarizes methods that do not require prior knowledge for inferring networks. The focus in this literature is on the mathematical and computational aspects involved in active learning of biological networks. In such work, generality is considered an advantage, and indeed, most of the methods do not limit themselves to a specific biological domain or specific type of network (regulatory, metabolic, etc.). Value is placed on a method's ability to infer a model from data with minimal or no prior knowledge and expert input. However, note that a method's ability to incorporate prior knowledge (without being completely dependent on the availability of prior knowledge) is also considered a strength.

We group the methods reviewed by the representations used for the models, starting with a review of work by Ideker et al. [1], who use the Boolean network representation. The conceptual simplicity of the Boolean network model makes it a good foundation upon which to build when discussing aspects of the other models. For this reason, our review of the work by Ideker et al. also serves as an introduction to concepts that are used in discussing other methods.

### Boolean networks

Ideker et al.[1] consider the task of learning a GRN. A node in the network may represent a gene or a stimulus, where a stimulus may be any factor that influences the network but is itself neither a gene nor a gene product. An edge in the network represents an influence of one gene on another, implicitly mediated by the product of the former gene, or the influence of a stimulus on a gene.

Inspired by the development of microarray technologies making global expression analysis feasible, Ideker et al. suppose that the available data consists of the expression levels of many (but not necessarily all) of the genes in the network. Each gene is represented by a variable, and the expression level of each gene is discretized into a Boolean value: 0 for low values indicating a repressed state, and 1 for high values indicating an activated state of the gene.

The Boolean variables representing the genes correspond to nodes in a directed acyclic graph (DAG) that forms the structure of a Boolean network. In a directed graph, nodes are connected by directed edges (usually represented by arrows when illustrated). We say that a directed edge points from a parent node to a child node. Hence, the parents of a node are all the nodes from which there are directed edges pointing directly to the node. A directed graph is a DAG if it contains no path that follows the directions of the edges and leads back to the node at which it started. The value of each node in the network is defined as a Boolean function of the values of its parents.

Ideker et al. developed a method for uncovering the structure and the Boolean functions of a network that describes the GRN of interest. The data available were gene expression measurements discretized to Boolean values. Global expression data from cells in an unperturbed steady-state yield a Boolean vector of 0s and 1s, with each element of the vector corresponding to a variable (gene or stimulus). There are many Boolean networks that are consistent with such a vector, but the underlying assumption is that one of these networks is the most accurate representation of the underlying biology. In order to uncover the most accurate network, additional data is needed (namely, expression data obtained from experiments in which the organism is perturbed). In such a perturbation, one or more of the genes or stimuli in the network are over- or under-expressed by the experimenter, effectively forcing the values of the manipulated variables to 1 (for over-expression) or 0 (for under-expression). Under the assumption that this perturbation does not change the dependencies that govern the unperturbed genes, the observations (Boolean vectors) produced by these experiments should also be consistent with the structure and functions of the Boolean network to be found. As the number of perturbation experiments grows, the number of Boolean networks that are consistent with all observations from all experiments decreases. The challenge of the task is not only to infer the most accurate network from a sequence of experiments but also to select the perturbations in a way that minimizes the number of experiments needed to infer the network.

Ideker et al. make explicit the division between these two tasks by describing two major components that make up their method: the “predictor,” which infers networks from data, and the “chooser,” which selects the next experiment in the sequence. The predictor takes as input a set of (Boolean) expression profiles and produces as output the set of networks that are both consistent with the profiles and have a minimal number of edges. That is to say that any network with fewer edges would necessarily be inconsistent with the data. See Ideker et al. [1] for a detailed description of the predictor's operation.

#### The chooser and entropy as an experiment selection criterion

The chooser takes as input the set of candidate Boolean networks produced by the predictor along with a set of queries describing candidate experiments that could be performed and chooses one of those queries. A query for an experiment consists of the list of variables to be perturbed as well as the Boolean values to which these variables are to be set in the experiment. Conceptually, the best experiment is one whose outcome will most discriminate between the available set of networks. An experiment whose outcome is consistent with all candidate networks is least informative, while an experiment whose outcome is only consistent with one network is most informative.

More concretely, given a set *M* of candidate Boolean networks and a set of candidate perturbation experiments, the chooser does the following. For each candidate experiment *e*, the network state predicted to result from *e* is computed according to each network. The network state is an assignment of all the Boolean variables (genes) in the network to values according to the Boolean functions defining the network. A given perturbation *e* yields a total of *X*_*e*_ distinct states (indexed by *x*) over the networks (1 ≤ *X*_*e*_ ≤ |*M*|), and the number of networks that predicted state *x* is denoted by $l_{x}$. The chooser selects the experiment *e* that maximizes the following entropy score *H*_*e*_:

$$H_{e}=-\sum_{x=1}^{X_{e}}\frac{l_{x}}{\left|M\right|}\log_{2}\frac{l_{x}}{\left|M\right|}.$$ (1)

*H*_*e*_ can be viewed as the expected decrease in entropy or, equivalently, a measure of the information gained in performing experiment *e*. To better see how this is a useful measure, consider two extreme cases: one in which all networks yield the same outcome (network state), and one in which all networks yield different states in response to experiment *e*. The value of *H*_*e*_ is at its minimum (*H*_*e*_ = 0) in the former case and at its maximum (*H*_*e*_ = log_2_ |*M*|) in the latter, reflecting the intuition that the former experiment is not informative and the latter is maximally informative. Consequently, it is not surprising that entropy is widely used as an experiment selection criterion, both in the context of active learning for biological networks [2–5] and in other domains of active machine learning.

#### The maximum difference criterion

Entropy is not the only criterion that might be used for selecting an experiment. Atias et al. [6] propose an alternative approach, which stems from the idea of distinguishing between alternate best-fit models. The maximum difference criterion selects the experiment that yields maximally different states for some pair of models in the set of best-fit models.

The general problem setup considered by Atias et al. [6] is similar to that considered by Ideker et al. [1]: the given data consists of Boolean vectors representing gene expression profiles under a variety of conditions, the underlying model to be learned is a Boolean network, and the aim of the experiment selection strategy is to arrive at a single model after as few experiments as possible. Whereas Ideker et al. used their predictor, Atias et al. use an algorithm by Sharan and Karp [7] for learning a Boolean network given experimental data. The Sharan and Karp algorithm solves an integer linear programming (ILP) optimization problem to find a Boolean network that is a best fit for the data available so far. Like the task faced by the predictor, this optimization problem typically has many equally optimal solutions.

Atias et al. use a modified version of the ILP to find a pair of models, *m*_1_ and *m*_2_, that solve the original optimization problem optimally and, additionally, find an experiment *e* that maximizes the difference between the network states that each model predicts for *e*. More precisely, let $x_{m_{1},e}$ and $x_{m_{2},e}$ be the binary vectors representing the states of the measurable variables predicted for experiment *e* by *m*_1_ and *m*_2_ respectively. The ILP Atias et al. use solves for the experiment *e* that optimizes

$$\begin{array}{l} \underset{e,m_{1},m_{2}}{\max}\;{\left\|x_{m_{1},e}-x_{m_{2},e}\right\|}_{1}\\ \;\text{s}.\text{t}.\;m_{1},m_{2}\;\text{are both optimal solutions of the Sharan and Karp ILP}. \end{array}$$ (2)

Here, since $x_{m_{1},e}$ and $x_{m_{2},e}$ are binary vectors, $\|x_{m_{1},e}-x_{m_{2},e}{\|}_{1}$ is the Hamming distance between them (the number of variables that are predicted to be different by *m*_1_ and *m*_2_).

#### Model evaluations

Ideker et al. evaluated their method by simulation, a common evaluation technique for active learning methods. They generated random networks to act as ground truth and simulated the unperturbed measurements, as well as the outcomes of all single-node perturbations. This set of measurements was the initial dataset given to the method. They then had the method choose a double-perturbation experiment, simulated the outcome of the experiment using the ground-truth network, and fed the result back for the method to iteratively propose the next experiment. This process was repeated until only a single network was consistent with the data.

Atias et al. compared the performance of their method to the entropy-based approach used by Ideker et al. Both simulated and real data evaluations were used for these experiments. Specifically, two models of signaling systems in humans were used as evaluation contexts: a Boolean model of epidermal growth factor receptor (EGFR) signaling constructed by Samaga et al.[8], which contains 112 molecular species (Boolean variables with associated functions), and a Boolean model model of interleukin 1 (IL1) signaling created by Ryll et al. [9] with 121 molecular species.

For simulated data experiments, the Boolean model (either EGFR or IL1) was used to generate Boolean vectors representing the measurements one would observe as a result of an experiment. Atias et al. generated a validation set for testing the performance of the methods. Any model that perfectly predicts the results of the validation set is considered an optimal model. The evaluation consisted of giving each method data from an initial pool of experimental results and measuring the number of additional experiments needed for each method to arrive at an optimal model.

For real data experiments, data published by Samaga et al. and Ryll et al. on phosphorylation levels under different cellular conditions in the two respective systems were used. For the EGFR system, the activity levels of 11 proteins were measured under 34 distinct conditions in Hep2G cells. For the IL1 system, the activity levels of 9 proteins were measured under 14 distinct conditions in primary hepatocytes. The evaluation consisted of starting each method with an initial subset of the available experiments and measuring the number of additional experiments selected from the pool of available experiments (34 for EGFR and 14 for IL1) that were needed to obtain a model as good as the one learned from all available experiments.

The maximum difference approach was found to perform better than the maximum entropy approach, and it was generally less sensitive to variation in the amount of initial data given and the number of unknown Boolean functions. Atias et al. reason that the maximum difference approach performed better because the entropy-based approach relied on estimation of the entropy from a sample of models, whereas the maximum difference approach was implemented using optimization over the full space of possible models.

The Boolean network representation used by Ideker et al. and Atias et al. is appealing in its simplicity and generality. However, one limitation is the representation's lack of robustness to noise in the data: observations are taken as binary values, and it is assumed that any model inconsistent with an observation must be rejected. In reality, measurements can be inaccurate and the outcomes of experiments may be influenced by uncontrolled factors. Thus, the inconsistency of a single observation with the model may not be sufficient reason to reject the model altogether. One approach to addressing the reality of noisy data is to explicitly model the outcomes of experiments as random variables. Probabilistic models do this, and one type of probabilistic model that has been applied to active learning of biological networks is the Bayesian network.

### Causal Bayesian networks

Several approaches to active learning using causal Bayesian networks have been developed [5, 10, 11]. A Bayesian network is a probabilistic graphical model that consists of a DAG whose nodes represent random variables **X** = *X*_1_,…,*X*_*p*_. A Bayesian network is similar to a Boolean network in that the edges in the graph represent direct dependence between variables. The difference is that whereas in a Boolean network, the value of a variable is governed by a Boolean function of its parents in the graph, in a Bayesian network, the value of a variable is governed by a conditional distribution of that variable given the values of its parents in the graph. These conditional distributions may be multinomial if the variable is in a discrete domain or may be some continuous distribution if the variable is in a continuous domain. Taken together, the local conditional distributions in the Bayesian network describe a joint probability distribution over all the variables of the network. For discrete variables, the joint probability that the variables take the values corresponding to a particular instantiation **x** = *x*_1_,…,*x*_*p*_ is

$$\mathbb{P}(X=x)=\prod_{i=1}^{p}\mathbb{P}(X_{i}=x_{i}|X_{\text{pa}(i)}=x_{\text{pa}(i)})$$ (3)

where **X**_pa(*i*)_ represents the subset of variables that are parents of *X*_*i*_ in the graph and **x**_pa(*i*)_ represents the corresponding values that these variables take in instantiation **x**. Similarly, for continuous variables, the joint probability density of the variables at **x** is

$$f_{X}(x)=\prod_{i=1}^{p}f_{X_{i}}(x_{i}|X_{\text{pa}(i)}=x_{\text{pa}(i)}).$$ (4)

Within the context of biological networks, the variables represent measurable quantities such as gene expression levels, as well as potential targets for perturbations such as gene silencing or overexpression.

There is extensive work on approaches to learning Bayesian networks from observational data; Daly et al. [12] provide a broad review. The main challenge in learning Bayesian networks is that of learning the graph structure from data. There are two main categories of approaches to Bayesian network structure learning: constraint-based methods, wherein conditional independencies (CIs) in the data are used to constrain the structure, and score search methods, wherein the space of Bayesian network structures is searched for a structure that has the best score according to some criterion.

Using observational data, Bayesian network structures can be inferred up to Markov equivalence. This concept of equivalence stems from the idea that a Bayesian network graph structure carries information about dependence and independence between variables. Consider a Bayesian network with three variables, *X*_1_, *X*_2_, and *X*_3_. A structure of *X*_1_ → *X*_2_ → *X*_3_ describes a situation in which *X*_1_ is independent of *X*_3_ given *X*_2_, but marginally, *X*_1_ and *X*_3_ are not independent. The exact same independence also holds in two other network structures: *X*_1_ ← *X*_2_ → *X*_3_ and *X*_1_ ← *X*_2_ ← *X*_3_. Another network structure, *X*_1_ → *X*_2_ ← *X*_3_, implies a different independence relationship: here, *X*_1_ and *X*_3_ are marginally independent but are not independent given *X*_2_. The dependencies and independencies are key to determining how well a particular Bayesian network structure fits observational data. Because observational data is limited in that we can derive associations from it but not causal relationships, it generally cannot be used to distinguish between network structures that capture the same dependence relationships, for example, between *X*_1_ → *X*_2_ → *X*_3_, *X*_1_ ← *X*_2_ → *X*_3_, and *X*_1_ ← *X*_2_ ← *X*_3_. For this reason, such sets of Bayesian networks are called equivalent.

Markov-equivalent structures can differ in the causal relationships they imply. For example, in the network *X*_1_ → *X*_2_ → *X*_3_, perturbing node *X*_1_ yields a change in *X*_2_ and *X*_3_, but in *X*_1_ ← *X*_2_ ← *X*_3_, perturbing node *X*_1_ does not affect the other two nodes. For this reason, it is difficult to infer causal relationships from observational data alone. There is existing work on predicting the effects of perturbations based on observational data in large-scale biological systems [13]. To uniquely determine a causal Bayesian network, however, perturbation experiments are needed.

According to a causal Bayesian network, a perturbation experiment changes the joint distribution of observations as follows. A perturbation at *X*_*i*_ affects only its descendants. More specifically, consider a perturbation or a randomized experiment *E* on a subset of variables **X**_*E*_: *E* ⊂ {1,…,*p*}. Effectively, under such an experiment, each variable *X*_*i*_ in the subset is made to follow some new distribution *U*_*i*_, and the intervention yields the joint probability distribution

ℙ(X=x|do(XE∼UE))=∏i∉Eℙ(Xi=xi|Xpa(i)=xpa(i))∏i∈Eℙ(Ui=xi) (5)

for discrete variables or the joint density

fX(x|do(XE∼UE))=∏i∉EfXi(xi|Xpa(i)=xpa(i))∏i∈EfUi(xi) (6)

for continuous variables, where do(**X**_*E*_ ∼ **U**_*E*_) denotes the perturbation [14]. Using this formulation of the expected effect of a perturbation on the probability distribution of observations, structure learning methods can be extended to take advantage of perturbation data. Cooper and Yoo [15] have presented a score for Bayesian networks of discrete variables, which has been extended to Bayesian networks of Gaussian random variables by Pournara and Wernisch [5]. More recently, Rau et al. [16] have presented an alternate method for scoring and efficiently searching for a best-fitting Gaussian Bayesian network from a mixture of observational and perturbation data for reconstructing regulatory networks.

#### Score-based active learning of causal Bayesian networks

Pournara and Wernisch [5] present a score-based active learning method for learning Bayesian networks. The scores they use [5, 15] capture the property of transition sequence (TS)-equivalence, which is an extension to perturbation data of Markov equivalence. For example, the set of three-node DAGs *X*_1_ → *X*_2_ → *X*_3_, *X*_1_ ← *X*_2_ ← *X*_3_, and *X*_1_ ← *X*_2_ → *X*_3_ form a Markov equivalence class. A perturbation at *X*_1_ would affect *X*_2_ and *X*_3_ in the first structure but not in the latter two; thus, {*X*_1_ → *X*_2_ → *X*_3_} and {*X*_1_ ← *X*_2_ ← *X*_3_,*X*_1_ ← *X*_2_ → *X*_3_} form two TS-equivalence classes with respect to the sequence of perturbations {*X*_1_}. The scores used by Pournara and Wernisch capture the property of TS-equivalence by always scoring TS-equivalent networks equally. Moreover, these particular scores are Bayesian marginal likelihoods, which, under the assumption of uniform priors over network structures, are proportional to the posterior probabilities of the networks given the data (see [5, 15] for details).

Pournara and Wernisch use an entropy criterion for experiment selection, wherein the scores are used to define the probability distribution over which the entropy is calculated, as follows. Let D represent all previously available observational and experimental data. From D, infer the partition of the space of possible Bayesian network structures into TS-equivalence classes, score them, and (for purposes of tractability) keep only the *n* top-scoring classes. Each class indexed by *q*,(1 ≤ *q* ≤ *n*) is associated with the posterior probability *P*_*q*_ of a network in the class; recall that all the networks in the class have the same score. Let *E* represent an experiment under consideration. Without knowing the outcome of *E*, but knowing which variables are perturbed in experiment *E*, the TS-equivalence class *q* will be further partitioned into *n*_*q*_ ≥ 1 TS-equivalence subclasses $C_{s}^{q},(1\leq s\leq n_{q})$. Pournara and Wernisch define a loss function for experiment *E* as

$$L(E,D)=\sum_{q=1}^{n}\sum_{s=1}^{n_{q}}P_{q}|C_{s}^{q}|\log|C_{s}^{q}|.$$ (7)

The most informative experiment to select is the one under which this loss is minimized. Note that this loss function computes the negative of the entropy of the TS-equivalence classes, weighted by the model scores.

Pournara and Wernisch evaluated their method using simulated data, which were generated by Bayesian networks. One part of the evaluation compared their method to a previous method for active learning of Bayesian networks by Tong and Koller [17]. The evaluation was performed using data generated by the Cancer network used by Tong and Koller, a discrete domain network with five variables. The evaluation showed that Pournara and Wernisch's method performed better in terms of *L*_1_ edge error, a measure of agreement with the true generative network. The measure is described in detail by Tong and Koller [17] and Murphy [18].

A larger-scale evaluation compared Pournara and Wernisch's approach to a random experiment selection strategy using data from randomly generated discrete-domain networks with up to 10 nodes and continuous-domain networks containing up to 60 nodes. The results showed that the active learning strategy identified more causal relationships correctly, especially when fewer experiments had been performed.

#### Constraint-based active learning of causal Bayesian networks

He and Geng [10] approach constraint-based active learning for Bayesian networks by considering the chain graph representations of equivalence classes. A chain graph is a graph that contains both directed and undirected edges, with the additional constraint that it contains no directed cycle. A Markov equivalence class of Bayesian networks can be represented by its essential chain graph, a graph that has the same skeleton as all the DAGs in the class. Each edge in the essential graph of a class is directed if and only if it is also directed in each DAG in the class. The task of identifying the causal DAG then becomes reframed as the task of correctly orienting all the edges in the essential chain graph. He and Geng show in a series of theorems how a randomized experiment (as described above) that perturbs a single node gives information about the orientation of undirected edges in the essential chain graph. They also show how similar orientation information can be obtained from quasi-experiments, in which a target variable may be affected by an experimenter but not perfectly isolated from its parents in the causal graph. Analogous to constraint-based graph structure learning, this yields a framework in which we can determine constraints on the graph structure by performing conditional independence tests on the observations that result from perturbations. In particular, a perturbation of the *i*-th variable *X*_*i*_ may yield one of *n* resultant edge orientations. An observation of orientation *s* ∈ {1,…,*n*} results in a reduced equivalence class containing $l_{s}$ DAGs.

For sequential experiments in which one variable is perturbed at a time, He and Geng propose two experiment selection criteria: a minimax criterion and an entropy criterion. The minimax criterion selects the variable *X*_*i*_ that minimizes $\max_{s=1}^{n}l_{s}$. That is to say that it selects the perturbation such that the least informative experimental outcome for that perturbation is most informative as compared to the least informative outcomes from perturbing other variables. The entropy criterion selects the variable *X*_*i*_ that maximizes the entropy:

$$H_{X_{i}}=-\sum_{s=1}^{n}\frac{l_{s}}{L}\log\frac{l_{s}}{L}$$ (8)

where $L=\sum_{s=1}^{n}l_{s}$. Selecting the variable to perturb that maximizes this score favors yielding small, equal-sized classes post-perturbation, which parallels the rationale behind the entropy criterion for Boolean networks. Note that, for large graphs, the number of possible postintervention orientations and the number of DAGs in each class make exact computation of the entropy infeasible, and the authors propose to randomly sample the space of DAGs to obtain an estimate of the quantity $\frac{l_{s}}{L}$.

He and Geng also present a batch algorithm for proposing a set of single-node perturbations that would identify the causal graph at once, rather than by sequentially selecting experiments. The algorithm exhaustively searches all possible combinations of variables to perturb to identify the smallest set that would guarantee that only one DAG satisfies all edge orientations, in every possible outcome. This approach is also computationally intensive, since it requires one to compute every possible edge orientation that results from every possible single-node perturbation and, in the worst case, it iterates over the power set of variables.

He and Geng evaluated their algorithms on simulated data using a five-node graph as a generating model and found that the sequential methods are more efficient (require fewer experiments to determine the graph structure) than the batch algorithm. Among the sequential methods, they found that the entropy criterion is most efficient.

Hauser and Bühlmann [11] similarly use the constraint-based framework. They present an alternative minimax criterion for sequentially selecting single-node perturbations. Denote by *ε* the set of perturbations performed thus far, each of which is a set *E* of indices of variables that are simultaneously perturbed in each intervention, and let *C*_*ε*_ denote the equivalence class of DAGs that is consistent with those prior perturbations and the associated observations. Note that the initial state in which only observational data are available can be denoted by *ε* = {∅}. The proposed criterion selects

$$X_{i}\;:\;i=\overset{p}{\underset{j=1}{\mathrm{argmin}}}\;\underset{G\in C_{\varepsilon }}{\max}\;\xi (C_{\varepsilon \cup \{\{j\}\}|G}),$$ (9)

where *ξ*(*C*) is the number of undirected edges in the essential chain graph of the equivalence class *C*, and *C*_*ε*∪{{*j*}}|*G*_ is the equivalence class that results when adding the outcome predicted by DAG *G* of the single-node perturbation of *X*_*j*_. The rationale behind this criterion is based on the idea that undirected edges in the essential chain graph of an equivalence class are a measure of uncertainty. This criterion picks the single-node perturbation that would minimize the maximal (worst-case) number of undirected edges in the post-perturbation class. Here, worst-case is considered in terms of the the causal graph *G* in the pre-perturbation class *C*_*ε*_ that would yield the most undirected edges post-perturbation if *G* were indeed the underlying causal model.

Hauser and Bühlmann also present a criterion for multiple-node interventions. This criterion computes a set of nodes

$$E=\underset{E^{\prime }\subset \{1,\ldots ,p\}}{\mathrm{argmin}}\;\underset{G\in C_{\varepsilon }}{\max}\;\omega (C_{\varepsilon \cup \{E^{\prime }\}|G}),$$ (10)

where *ω*(*C*) denotes the size of the largest undirected clique in the essential chain graph of class *C*. Similar minimax reasoning behind this criterion applies, with the additional intuition that large undirected cliques represent high uncertainty about the causal flow between the nodes in the clique. Hauser and Bühlmann also prove that maximally reducing the clique number after each perturbation yields full identifiability of the DAG with a minimal number of (multiple-node) perturbation experiments.

Hauser and Bühlmann evaluate their algorithms for optimizing these two objectives on simulated data from 4,000 randomly generated causal DAGs with *p* ∈ {10,20,30,40} nodes. Their results show that the single-node criterion recovers the DAG while perturbing the fewest number of nodes, while the multiple-node criterion recovers it using the fewest number of perturbation experiments (although, since multiple nodes are perturbed in each experiment, more nodes are perturbed in total).

### Probabilistic temporal Boolean network

Dehghannasiri et al. [19] apply the mean objective cost of uncertainty (MOCU) criterion for optimal experiment selection. Unlike other experiment selection criteria, MOCU is meaningful in the context of applying an intervention that may have undesirable outcomes. The motivation for this context is that of altering pathological phenotypes, a task of major interest in translational genomics. The objective is not simply to select experiments that are informative but to select experiments that would inform us in such a way that the probability of producing undesirable outcomes when using the model to select an intervention is minimized. MOCU can be generally computed when there is a distribution over states, some of which are undesirable.

Dehghannasiri et al. use probabilistic Boolean networks that model gene activity over time. Recall that Ideker et al. [1] and Atias et al. [6] use the Boolean network to model the steady state of the network directly. In contrast, Dehghannasiri et al. use the Boolean network as a discrete-time model of a GRN. In this context, the state of a gene at time *t* is a Boolean function of the states of its parents in the network at the previous time point *t*−1. The model is made probabilistic by further assuming that each variable has a small probability to take the value opposite to that which is governed by the Boolean function at each time point. This description of network dynamics is used to define the transition probabilities in a Markov chain and arrive at a steady-state distribution that describes the probability that a given gene is active at any given moment in the steady state [20].

Given the goal of applying an intervention to the GRN, assume that there are some undesired states (i.e., we prefer that some specific genes be activated/repressed). This is translated to a cost associated with seeing a given gene activated or repressed. Given these costs and a probabilistic steady-state distribution, a cost is associated with the steady state by taking a weighted average over the probabilities of the undesired states in the steady-state distribution. Suppose that we have a prior distribution over GRNs and a given intervention. We can compute the expected cost of performing the intervention as a weighted average according to the GRN distribution over the steady state costs of the GRNs post-intervention. Dehghannasiri et al. call the intervention that minimizes this expected cost the intrinsically Bayesian robust intervention. The MOCU is then defined as the expected cost of applying the robust intervention over all networks.

The uncertainty about the GRNs is represented in terms of parameters about which we are uncertain, and we call the set of all possible realizations of such parameters an uncertainty class of GRNs. Starting with a prior uncertainty class of GRNs, each potential experiment reduces the uncertainty class of GRNs. Note that Dehghannasiri et al. assume that each experiment can determine an unknown parameter, which in the case of a GRN means determining whether one particular gene up-regulates or down-regulates another, and they do not specify any particular lab technique to which this corresponds. The experiment to select is then defined as that which minimizes the MOCU of the reduced uncertainty class.

Dehghannasiri et al. tested their approach in simulations in which data were generated from both randomly generated networks and a 10-gene regulatory network of the mammalian cell cycle and a 6-gene network of p53, a tumor suppressor gene. In both cases, they considered the task when 2, 3, 4, or 5 regulatory relationships were unknown. The results with the randomly generated networks showed that the less uncertain the initial network, the larger the gain of selecting an experiment using MOCU over a random selection, where gain was measured in terms of the cost of applying the resultant robust intervention to the true network. Another evaluation metric used was the rate of success and failure of the intervention preventing undesirable states, which showed that MOCU led to a better success rate. The experiments with the mammalian cell cycle network and the p53 network showed similarly a general improvement in intervention success when using MOCU.

### Differential equation models

Another approach to modeling a biological network is that of differential equation models of expression levels. Steinke et al. [4] represent the expression levels of the *N* genes in a model at time *t* by a vector **x**(*t*) ∈ ℝ^*N*^, which is governed by the stochastic differential equation (SDE)

$$dx(t)=\underset{\text{dynamics}}{\underset{︸}{g(x(t))}}dt-\underset{\text{perturbation}}{\underset{︸}{e(t)}}dt+\underset{\text{noise}}{\underset{︸}{dW(t)}}$$ (11)

where **g**: ℝ^*N*^ → ℝ^*N*^ describes the nonlinear system dynamics of gene regulation, **e**(*t*) represents perturbation caused by an experiment, and *d***W**(*t*) is white noise. Tegnér et al. [21] use an ordinary differential equation (ODE) variant of the model without the *d***W**(*t*) term. In both formulations, the goal is to uncover the unknown system dynamics **g**.

The problem of recovering **g** directly is underspecified. A common approach is to restrict the problem to that of discovering a linear approximation of the dynamics around the system's unperturbed steady state instead. Given that the system settles in a steady state **x**_0_ subject to the absence of perturbation (that is, **e**(*t*) ≡ **0**), linearization of the system about that point yields a linear model of a new steady state **x**_**e**_ that would result from applying a constant perturbation **e**(*t*) ≡ **e**:

$$e=A(x_{e}-x_{0})+\varepsilon .$$ (12)

Here, **A** is the matrix describing the local linear system dynamics, and *ε* is a noise term from the stochastic model, which Steinke et al. assume to be component-wise independent identically distributed (IID) Gaussian with variance *σ*^2^. Nonzero *a*_*ij*_ entries correspond to edges in the gene regulatory network. Both Tegnér et al. and Steinke et al. take a common approach and focus on the steady-state differences **x** = **x**_**e**_ − **x**_0_. This notation simplifies Eq (12) to

e=Ax+ε. (13)

The task of learning the gene regulatory network is therefore translated to the task of inferring **A** from observations of expression level differences **x** subject to perturbations **e**.

#### Sparse Bayesian model

Steinke et al. employ a Bayesian approach. They treat **A**, **e**, and **x** from Eq (13) as random variables, thus converting the task of learning the matrix **A** to that of estimating the posterior distribution ℙ(**A**|**E**,**X**) of **A** given data consisting of the known perturbations **E** and measurements **X**. To estimate this probability, Bayes' rule is employed:

ℙ(A|E,X)∝ℙ(E|A,X)ℙ(A). (14)

The distribution ℙ(**E**|**A**,**X**) is given by the linear model described in Eq (13): For a given **A** and **x**, adding the Gaussian noise term *ε* yields a Gaussian centered at **Ax** with covariance matrix *σ*^2^**I**, or equivalently, $\left(E|A,x)∼N(\mathrm{Ax},\sigma^{2}I\right)$. GRNs are known to be sparsely connected, typically averaging less than two transcriptional regulators per gene [22]. To reflect this, the prior distribution of **A** is taken to be a Laplace sparsity prior,

$$f_{A}(A)=\prod_{i,j}f_{A_{ij}}(a_{ij}),\;f_{A_{ij}}(a_{ij})=\frac{\tau }{2}e^{-\tau |a_{ij}|}.$$ (15)

The experiment selection criterion used by Steinke et al. is an information-theoretic one, conceptually similar to the entropy-based criteria described in methods above. The main principle at work is the estimation of the change in the posterior distribution of **A** that the next experiment (to be selected) would yield. Denote the data available thus far by D and denote the posterior given data D by ℙ(A|D)=Q(A). Let (e_*_,x_*_) represent an experiment and its outcome, and denote the posterior after the additional experiment by $Q^{\prime }(A)=\mathbb{P}(A|D\cup \{(e_{*},x_{*})\})$. A measure of the uncertainty in *Q* is the differential entropy $E_{Q}[-\log Q]$, hence, given the posterior *Q*′, the change in uncertainty due to observing the experiment is the Kullback–Leibler divergence [23] between *Q*′ and *Q*,

$$S(e_{*},x_{*}|D)=D_{KL}[Q^{\prime }\|Q]=E_{Q^{\prime }}[\log Q^{\prime }-\log Q].$$ (16)

The choice of model in this setting enables efficient computation of the information gain for a specific experimental outcome (e_*_,x_*_). The outcome of an experiment, however, is not known before it is performed. The current model, however, can yield a distribution *Q*(X_*_|e_*_) for the outcome as a random variable X_*_ predicted based on e_*_, according to the current distribution *Q*(**A**). This distribution is used to obtain the expected information gain

$$S(e_{*}|D)=E_{Q(X_{*}|e_{*})}S(e_{*},X_{*}|D)=E_{Q(X_{*}|e_{*})}D_{KL}[Q^{\prime }\|Q]$$ (17)

which is used as the criterion for selecting an experiment. Given a list of candidate perturbations to perform, the method identifies the perturbation e_*_ which maximizes this score.

Steinke et al. evaluated the method on simulated data generated by randomly constructed networks with nonlinear Hill-type kinetics. They also evaluated the method using the Drosophila segment polarity network [24].

#### Reverse engineering a linear gene network model

Tegnér et al. [21] approach the task of reconstructing a GRN as that of fitting a linear gene network model. They take the approach of approximating the ODE by a linear function around the unperturbed steady state

e=Ax (18)

where **e** is a vector representing the perturbation, and **x** is a vector representing the observed change from the steady state of gene expression levels. In order to fit this model, the system under study must be observed across a variety of deviations from the steady state.

Tegnér et al. present a reverse-engineering algorithm that ensures that changes are observed in all genes of interest (in the context of this review, “perturbation” means that a gene was directly manipulated by an experiment, while “change” encompasses all changes of expression [either from direct perturbation or indirectly as a result of perturbation in a different gene]; Tegnér et al. use slightly different terminology). Experiment selection is broken up into several stages. Initially, when no data is available, two measurements are performed: one of the unperturbed steady state and another of the steady state resulting from the perturbation of a single randomly selected gene. Presumably, the outcome of perturbing this gene will yield changes of varying degree in other genes as well.

The second phase ensures that the expression level of every gene has been changed beyond a prespecified threshold in some experiment, either from direct perturbation or indirectly as a consequence of perturbing another gene. The algorithm dictates that the next gene to be perturbed (of the genes that have not yet been perturbed) is the one that has shown the least change in any of the previous experiments. This is repeated until all genes have shown a change beyond the prespecified threshold.

In the third phase, genes to perturb are selected iteratively until the model matrix *A* is reconstructed. For each previously done experiment and each gene, a solution to Eq (18) is produced that estimates the inputs to the gene (a gene *j* is an input to gene *i* when the corresponding entry in *A* is nonzero). Next, for each gene *i* and each gene *j*, the variance of the level of *j* when it is taken as input to gene *i* across all solutions is computed, and a ranked list of genes *j* is produced for each gene *i*. The ranking for each gene *j* is summed to produce a single number that represents how much that gene varies across solutions in a relative sense. The highest ranking genes are selected for perturbation experiments, and the process is repeated until sufficient perturbations have been performed to reconstruct *A*.

Tegnér et al. tested their method in simulations on a model of the segmentation polarity network in *Drosophila* and showed ability to recover the network structure even when the simulating model is nonlinear, in spite of the fact that the inferred model is linear.

#### A difference criterion for differential equation models

Another experiment selection criterion that has been proposed for differential equation models is based on the idea of maximizing differences between the outcomes of an experiment under competing models. The maximum difference criterion of Atias et al. [6] discussed above parallels a similar approach for ODE models by Mélykúti et al. [25]. Mélykúti et al. focus abstractly on the problem of designing the experiment that would distinguish between a pair of ODE models. Specifically, experimental design consists of specifying the values of a subset of input variables that can be manipulated experimentally. Given that the system has a set of output variables that are measured and two rival models that need to be distinguished, the approach designs an experiment that maximizes the *L*_2_-norm of the difference between the predicted outputs of the two models considered.

### Purely structural model

Ud-Dean and Gunawan [26] take an approach to learning GRNs in which only the network structure is explicitly modeled. This is unlike Bayesian and Boolean networks, which explicitly model the expression levels or abundances of genes or proteins that correspond to nodes in the graph structure. Ud-Dean and Gunawan make the assumption that a knockout (KO) of a gene causes differential expression in all genes that are downstream of it (its descendants) in the network. This assumption is used to eliminate network structures that are inconsistent with data.

Taking in gene expression profiles under various single KO conditions as initial data, the algorithm determines ancestry relationships among all the genes in the network. The ancestries are determined by following the assumption above: if gene *j* shows differential expression between the wild-type and a knockout of gene *i*, then gene *i* is taken to be an ancestor of gene *j* in the network. Differential expression is determined by performing a two-sample *t* test on expression level measurements. These ancestry relationships are then translated into an “upperbound” network and a “lowerbound” network. The lowerbound contains only edges that are necessary to satisfy the ancestry relationships, meaning that removing any edge from the lowerbound network would make some node *i* a non-ancestor of node *j* when it was determined that *i* is an ancestor of *j*. The upperbound contains all edges that can possibly satisfy the ancestry relationships, meaning that adding any edge to the upperbound network would make some node *i* an ancestor of node *j* when it was determined that *i* is not ancestor of *j*. Edges that are present in the upperbound network and are absent in the lowerbound network are then considered uncertain.

The task of the active learning procedure is to test hypotheses about the presence of uncertain edges. The query that the procedure constructs consists of a collection of multiple-KO experiments, characterized by a background deletion $V_{KO}^{*}$ of one or more genes and a set of genes I^*^ to be individually deleted in the presence of the background deletion. For example, referring to genes by numbers, if $V_{KO}^{*}=\{1,3,5\}$ and I^*^ = {2,4}, one would perform the background triple-knockout {1,3,5} and the two quadruple-knockouts, {1,2,3,5} and {1,3,4,5}.

The purpose of using a background KO is to eliminate indirect paths from genes *i* to *j* when testing a hypothesis about the presence of an uncertain edge from *i* to *j*. The expression of gene *j* is measured subject to the background KO and subject to the KO of gene *i* in conjunction with the background KO, and a two-sample *t* test is used to determine differential expression of *j* between these KO conditions. Rejection of the null hypothesis indicates the presence of the edge from *i* to *j*, and the edge is consequently added to the lowerbound, while failure to reject the null hypothesis indicates the absence of the edge, and the edge is consequently removed from the upperbound.

The criterion that Ud-Dean and Gunawan use maximizes the number of uncertain edges that can be verified using a particular background KO. They apply some constraints to reduce the complexity of the optimization problem and use a genetic algorithm to solve it, meaning that the background KO's selected might not be perfectly optimal. Some other practical considerations include constraining the number of KOs that can be selected, as well as restricting the algorithm from selecting KO combinations that are known to be lethal. Simulations showed that the method was able to recover a GRN more faithfully than one can from the set of all double-KOs, using significantly fewer experiments. Moreover, the GRN was perfectly recovered in simulations with no noise in the data (that is, ones where differential expression was correctly identified) and the assumption about differential expression happening downstream of a perturbation knockout held.

## Active learning with prior knowledge

This section summarizes methods that extensively incorporate prior knowledge about the biological system of interest in modeling the networks and designing experiments. While reliance on prior knowledge sacrifices generality, it often facilitates more accurate modeling of biological systems when targeting specific questions about those systems. Each work summarized here aims to answer a specific question in a specific biological domain. As a result, the type of model used, prior knowledge incorporated, and knowledge discovered in each method is different.

### Validation and refinement of gene regulatory models in yeast

Yeang et al. [3] aimed to elucidate regulatory relationships among genes and proteins in yeast (*Saccharomyces cerevisiae*). They combined data from prior knowledge and data from knockout experiments in order to produce a network representation of the regulatory relationships, the physical network model. In a physical network, nodes represent genes and proteins, and the edges between them represent induction/repression for protein–DNA interaction and activation/inhibition for protein–protein interactions. In this setting, multiple networks can provide equally probable competing explanations for the data available, and the role of active learning is to design additional knockout experiments that would resolve such uncertainty.

The prior knowledge Yeang et al. use comes from databases of protein–protein and protein–gene interactions. Specifically, they used promoter-binding interactions for transcription factors measured by Chromatin immunoprecipitation microarray (ChIP-chip) [27] and protein–protein interactions recorded in the Database of Interacting Proteins (DIP) [28]. This data is combined to build a skeleton graph of possible interactions between proteins and genes. In the skeleton graph, protein–protein interactions are undirected. Protein–gene interactions are directed from the proteins to the genes, since these are promoter-binding interactions. The resulting skeleton graph constitutes a summary of potentially relevant interactions. Yeang et al. [29] developed a method that takes this information and combines it with experimental data (specifically, mRNA expression profiles from individual gene-deletion experiments) to produce an output network that contains a subset of the skeleton graph's edges and in which all edges are directed and have signs (are annotated as inducing/repressing for protein–DNA interactions and activating/inhibiting for protein–protein interactions).

To infer a fully specified physical network from the skeleton graph, prior knowledge, and experimental data, Yeang et al. use a factor graph [30], a type of probabilistic graphical model, to formalize the problem mathematically. In a factor graph, a joint probability distribution is defined over a collection of variables **Y** = *Y*_1_,…,*Y*_*p*_ (taking values in some space Y) in terms of a collection of *factors* (also known as potential functions). Each factor is a function $\phi_{i}:Y_{i}\to \mathbb{R}$ of a subset of the variables **Y**_*i*_ ⊂ **Y**. The joint probability of a particular assignment of all variables to values **y** is then defined as proportional to the product of all factors evaluated at **y**:

$$\mathbb{P}(y)=\frac{1}{Z}\prod_{i}\phi_{i}(y_{i})\;\text{where}\;Z=\sum_{y\in Y}\prod_{i}\phi_{i}(y_{i}).$$ (19)

In order to frame the problem of finding a physical network as a factor graph, Yeang et al. define binary variables that represent edge presence and direction for every possible edge (based on the skeleton graph); they define factors that correspond to evidence for protein–gene edge presence from chIP location analysis p-values, factors that correspond to evidence for protein–protein edge presence based on expression profile reliability (EPR) and paralogous verification method (PVM) [31] tests; and they define factors that correspond to each knockout experiment (representing the constraint that there must be a causal path from a knockout to its observed effects). Once the factor graph is defined, the maximum a posteriori (MAP) physical network is produced using the max-product algorithm, an efficient algorithm for finding the MAP variable assignment of a factor graph [30]. When there are multiple output networks that are equally most probable, additional experiments are necessary to arrive at a single network.

To efficiently select experiments, Yeang et al. use expected information gain as the criterion for selecting experiments. The information gain is computed as follows: let *M* represent the set of most-likely networks that were inferred from available data, and let *X* be the set of possible expression changes (outcomes) due to an experiment *e*. The information gain is a difference between the entropies of the model space before and after an experiment:

$$I(M;X,e)=-\sum_{m\in M}\mathbb{P}(m)\log_{2}\mathbb{P}(m)+\sum_{m\in M,x\in X}\mathbb{P}(m,x,e)\log_{2}\mathbb{P}(m|x,e)$$ (20)

The factor graph is used to compute the probabilities to obtain the information gain for each potential deletion experiment *e*, and the experiment that maximizes this quantity is selected. This follows the common theme present throughout this review, wherein the experiment is selected based on the decrease in model-space entropy it is expected to yield.

In the process of refining and validating the GRN of yeast, Yeang et al. used factor graph inference to account for 273 individual gene-deletion experiments, wherein gene expression profiles were measured by microarray. The effects of the knockouts were explained by paths in the physical network produced by the factor graph inference; these paths covered 965 interactions, of which 194 effects were uniquely determined and 771 were ambiguous. An example of an ambiguous interaction that they present is the explanation of up-regulation of promoters bound by Msn4p in a *swi*Δ knockout: "[O]ne scenario is that *SWI4* activates *SOK2* and *SOK2* represses *MSN4*, whereas another is that *SWI4* represses *SOK2* and *SOK2* activates *MSN4*." Paths with such ambiguous interactions were partitioned into 37 independent network models (numbered 1–37), and all remaining unambiguous interactions were put into one model (numbered as Model 0). Yeang et al. found confirmation in the literature for 50 of the 132 interactions in Model 0. To validate the ambiguous models, the information theoretic criterion was used to produce a ranked list of knockout experiments and found that 3 of the top 10 highest ranked experiments were targeting Model 1. For this reason, they focused on Model 1 in their analysis and reported that the four highest-ranking knockouts for Model 1 were conducted (namely *sok*2Δ,*yap*6Δ,*hap*4Δ, and *msn*4Δ). Using the data from these experiments, factor graph inference fully disambiguated Model 1 by disambiguating 60 protein–DNA interactions.

### Adam the Robot Scientist

King et al. [32] aimed to identify the genes that encode orphan enzymes in the aromatic amino acid (AAA) synthesis pathway in *S*. *cerevisiae*. Orphan enzymes are those for which the encoding genes are not known. These enzymes are nevertheless believed to be present because they catalyze reactions that are thought to occur in the given organism. King et al. combine knowledge of the yeast metabolic pathway and a database of genes and proteins involved in metabolism to design experiments that test whether a particular gene codes for a particular enzyme. The role of active learning in this setting is to select experiments in a manner that minimizes the total cost of identifying the genes that code for all the orphan enzymes in the pathway.

To accomplish this task, King et al. designed and implemented Adam the Robot Scientist. Adam was remarkable in that it was more than just a software system that selects experiments: it autonomously controlled lab equipment that conducted the proposed experiments, recorded the results, and used the results to update the model without any human intervention at any point of the active learning loop (beyond maintenance of the equipment and replenishment of supplies for experiments). Adam combined prior knowledge of the AAA pathway and results from its own experiments to iteratively perform auxotrophic growth experiments until all orphan enzymes were associated with their genes.

The prior knowledge that Adam used consists of a model of yeast metabolism and a database of genes and proteins involved in metabolism. This knowledge was encoded manually into a Prolog program. The resultant model could then be used to infer how the AAA pathway would behave in the absence of a particular enzyme in a particular growth medium. The uncertain component of the model is the assignment of orphan enzymes to genes, and the goal was to find the correct assignment.

Auxotrophic growth experiments are a common technique for discovering the details of a metabolic pathway. In such an experiment, a deletion mutant is grown in a specified growth medium, and growth is measured. For example, to test whether a gene codes for a specific enzyme, one would grow mutants where that gene is deleted. When grown in a medium that contains the metabolite product of the enzyme's reaction, the cells should grow as normal, but growth in the absence of the metabolite should be hindered. The outcome of each experiment can be viewed as either positive or negative, depending on whether growth was hindered. Therefore, to design an auxotrophic growth experiment, a decision must be made about what gene is to be deleted and what metabolites the growth medium should contain.

The experiments in this study were designed using a cost function. Many of the approaches discussed in this review focus on minimizing the number of experiments performed, which can be viewed as minimizing a cost function where each experiment has equal cost. King et al., however, use a cost function that also factors in the monetary cost of the materials used. The cost function calculates the expected cost of narrowing down from a set of mutually exclusive hypotheses to a single hypothesis using a set of possible experiments, where each hypothesis is an assignment of a gene to an enzyme. The idea is that each experiment *e* divides a set of mutually exclusive hypotheses *M* into two subsets, *M*_[*e*]_ and $M_{\bar{\left[e\right]}}$, representing, respectively, those hypotheses that are consistent with a positive outcome of *e* and those that are consistent with a negative outcome. Given a set of hypotheses *M* and a set of possible experiments *E*, the minimum expected cost of arriving at the single correct hypothesis by experimentation is defined by the recurrence:

EC(∅,E)=0 (21)

EC({m},E)=0 (22)

$$EC(M,E)=\underset{e\in E}{\min}\left[C_{e}+\mathbb{P}(e)EC(M_{\left[e\right]},E-e)+(1-\mathbb{P}(e))EC(M_{\bar{\left[e\right]}},E-e)\right]$$ (23)

where *C*_*e*_ is the cost of experiment *e* and ℙ(*e*) is the probability that the outcome of *e* is positive. Eq (21) represents the case in which we have no plausible hypotheses remaining and the expected cost is therefore 0, since no more experiments are needed; and Eq (22) represents the case where we have successfully narrowed down our hypothesis set to a single hypothesis *m*, and the expected cost is again 0, since no additional experiments are needed. The probability of a positive experiment outcome is computed as the sum of the probabilities of the hypotheses that are consistent with that outcome. It is generally intractable to compute the expected cost exactly, because every possible sequence of experiments with both positive and negative outcomes for each experiment would need to be considered. Consequently, the system uses an approximation to this cost function [33]

$$EC(M,E)\approx \underset{e\in E}{\min}\left[C_{e}+\left(\frac{1}{|E|-1}\sum_{e^{\prime }\in E-\{e\}}C_{e^{\prime }}\right)\left(\mathbb{P}(e)J_{M_{\left[e\right]}}+(1-\mathbb{P}(e))J_{M_{\bar{\left[e\right]}}}\right)\right]$$ (24)

$$\text{where}\;J_{M}=-\sum_{m\in M}\mathbb{P}(m)\lfloor \log_{2}\mathbb{P}(m)\rfloor .$$ (25)

In this approximation, the term $\frac{1}{|E|-1}\sum_{e^{\prime }\in E-\{e\}}C_{e^{\prime }}$ is the average cost of an experiment in *E* excluding *e* (the cost *C*_*e*_ of *e* is added directly), and the term $\mathbb{P}(e)J_{M_{\left[e\right]}}+(1-\mathbb{P}(e))J_{M_{\bar{\left[e\right]}}}$ is an estimate of the number of future experiments that would be performed after *e*. The estimate for the number of future experiments is based on Shannon's source coding theorem [34]. Given the task of designing a code to transmit a message, where the probability of encountering a message *i* is ℙ(*i*), the length of a binary code for *i* in an optimal coding scheme is −log_2_ℙ(*i*). If we consider the outcomes of experiments to be analogous to bits in a coding scheme, where the outcomes of a series of experiments “encode” each hypothesis by eliminating all other hypotheses, then we get that the number of experiments needed to eliminate all hypotheses except *m* is at most ⌈−log_2_ℙ(*m*)⌉, where the quantity is rounded up to ensure an integer number of trials. *J*_*M*_ in Eq (25) is simply the probability-weighted average of this quantity for any hypothesis in *M*, and it corresponds to the entropy of the set of hypotheses. Adam used the approximation in Eq (24) to select the next experiment *e* at each iteration of the process of narrowing down the hypothesis space.

King et al. [35] document development of the system, including testing of the cost function against previously collected data on known-function genes. The tests showed that the system outperformed both a random strategy and a "cheapest trial" strategy—one in which the next cheapest available experiment is selected at each iteration, without consideration of the hypotheses it rejects or the projected cost of future experiments. Additionaly, performance was comparable, in terms of cost and number of trials, to that of humans (computer science and biology graduate students).

King et al. [32] report that Adam formulated and tested 20 hypotheses concerning gene encodings of 13 orphan enzymes. Twelve hypotheses with no previous evidence were confirmed. A hypothesis about one enzyme was confirmed experimentally. A literature survey confirmed strong empirical evidence for the correctness of six of the hypotheses (i.e., the enzymes were not orphans in retrospect).

### Regulatory mechanisms downstream of a signaling pathway

Szczurek et al. [2] aim to reveal mechanisms of transcription regulation in the cell (specifically, to match transcription factors in a signaling pathway to the genes they regulate based on expression profiles under gene knockout and overexpression experiments). They identify two major challenges that cause ambiguity in inferring such relationships from expression profile data. One issue occurs when a transcription factor is inactive in all experiments conducted. In this case, its targets cannot be revealed. The second issue occurs when two transcription factors in two distinct pathways are both activated in a set of experiments. In this event, genes that are regulated by either one of the transcription factors erroneously appear to be coregulated by both, even if there is no coregulation. The role of active learning in this setting is to design experiments that reduce such ambiguities.

Szczurek et al. [2] introduce model expansion experimental design (MEED), an active learning algorithm for the task. MEED requires knowledge of the signaling pathway as input in the form of a predictive logical model. The process MEED uses has two parts: an expansion procedure that identifies regulatory modules and an experimental design step that uses the model to propose a collection of experiments that would disambiguate any ambiguous modules.

A regulatory module is a group of genes such that all the genes in the group are regulated by the same transcription factor. A simplifying modeling assumption that Szczurek at al. make is that each gene is regulated by only one transcription factor. A regulatory module is considered ambiguous when there are multiple possible transcription factors that may regulate a gene in a manner that is consistent with the data.

The matching of transcription factors to genes is done by the expansion procedure. The procedure uses the model of the signaling network and data from previous experiments. The type of experiment conducted may involve the manipulation (deletion or overexpression) of a gene in the signaling pathway and some input stimulus. The data measured in the experiment represent levels of gene expression. The signaling network model is used to predict the activation of the downstream genes in the network based on the stimulus and manipulation. Some of these downstream genes code for potential transcription factors. The expansion procedure searches for genes whose activation patterns are statistically associated with the predicted activation patterns of the potential transcription factors. Thus, a set of genes that matches the activation pattern of a particular potential transcription factor is identified as a regulatory module that is regulated by that transcription factor. When there are multiple transcription factors that show the same activation patterns throughout all experiments observed, a regulatory module that matches any one of the factors consequently matches the others in that group as well. When there are multiple transcription factors that match a regulatory module, the module is ambiguous in the sense that it is unknown which transcription factor regulates which genes in the module. In order to disambiguate ambiguous regulatory modules, the goal of MEED is to propose a set of experiments that maximizes the number of different activation patterns exhibited by all potential transcription factors and, moreover, to do so using the smallest possible number of experiments.

The principle that the experimental design follows is that of maximizing entropy. Specifically, for a set of experiments *E*, the calculation is as follows. A set of transcription factors that show the same activation patterns and regulate genes in the same manner form a regulatory program. Let *r* be the number of possible regulatory programs. A set of experiments *E* partitions the space of possible regulatory programs into *n* disjoint blocks with *n*_*b*_,(1 ≤ *n*_*b*_ ≤ *r*) programs in each block indexed by *b*,(1 ≤ *b* ≤ *n*). Each block is consistent with a different potential set of gene expression profiles as outcomes of the set of experiments. The entropy of this partition is

$$H(E)=-\sum_{b=1}^{n}\frac{n_{b}}{r}\log\frac{n_{b}}{r}.$$ (26)

Ideally, the set of experiments *E* selected is one that maximizes this entropy. However, because it is intractable to consider all possible sets of experiments *E*, MEED instead constructs a list of experiments *e*_1_,*e*_2_,*e*_3_,… in a greedy fashion. The first experiment *e*_1_ is the single experiment that maximizes the entropy *H*({*e*_1_}) for sets of one experiment. Each subsequent experiment *e*_*j*_ is chosen such that it maximizes the entropy of the list *H*({*e*_1_,…,*e*_*j*_}) given that the the selections of the preceding experiments *e*_1_,…,*e*_*j*−1_ have been fixed. MEED adds experiments to the list in this manner until either all potential experiments are listed or the entropy cannot be further maximized. The top several experiments in the resulting list can then be conducted in the laboratory, with practical considerations dictating the precise number of experiments.

MEED is unlike most other active learning methods discussed in this review in that it is designed to select sets of experiments rather than a single experiment at a time. If the active learning procedure selects one experiment at a time, this necessitates performing the experiments sequentially, performing a single experiment before feeding its results to the method to select the next one. In practice, performing multiple experiments in parallel rather than sequentially can be more time- and cost-efficient. Szczurek et al. tested whether it is actually necessary to score experiments jointly as MEED does or whether selecting top-ranked (by *H*({*e*})) individual experiments suffices for parallel experimentation. Using a simulation, they found that the MEED strategy performs much more efficiently.

In quantifying the performance of compared methods, Szczurek et al. measure the fraction of undistinguished pairs (FUP) for a set of experiments *E*. This is defined formally as

$$FUP(E)=\frac{\sum_{b=1}^{n}n_{b}(n_{b}-1)}{r(r-1)}$$ (27)

where *n*_*c*_, *C*, and *r* are as above. This score computes the fraction of regulatory program pairs that were not distinguished by the set of experiments out of the total set of regulatory program pairs; a value of 0 corresponds to distinguishing all regulatory programs, while a value of 1 corresponds to distinguishing none.

Szczurek et al. applied MEED to the yeast signaling pathway responsible for cellular response to hypersonic and pheromone triggers [2]. They report that MEED outperforms other methods (including the method developed by Ideker et al. [1]) when tested with real data from 25 experiments previously reported in the literature. Performance was evaluated by plotting a learning curve of FUP score over number of experiments performed, with MEED showing consistently lower (hence, better) FUP scores throughout the learning process. As additional validation, having provided MEED with all available experimental results, they examine the regulatory modules identified by the expansion procedure and identify corroborating evidence for their validity in the literature.

## Conclusions

**Fig 2:**
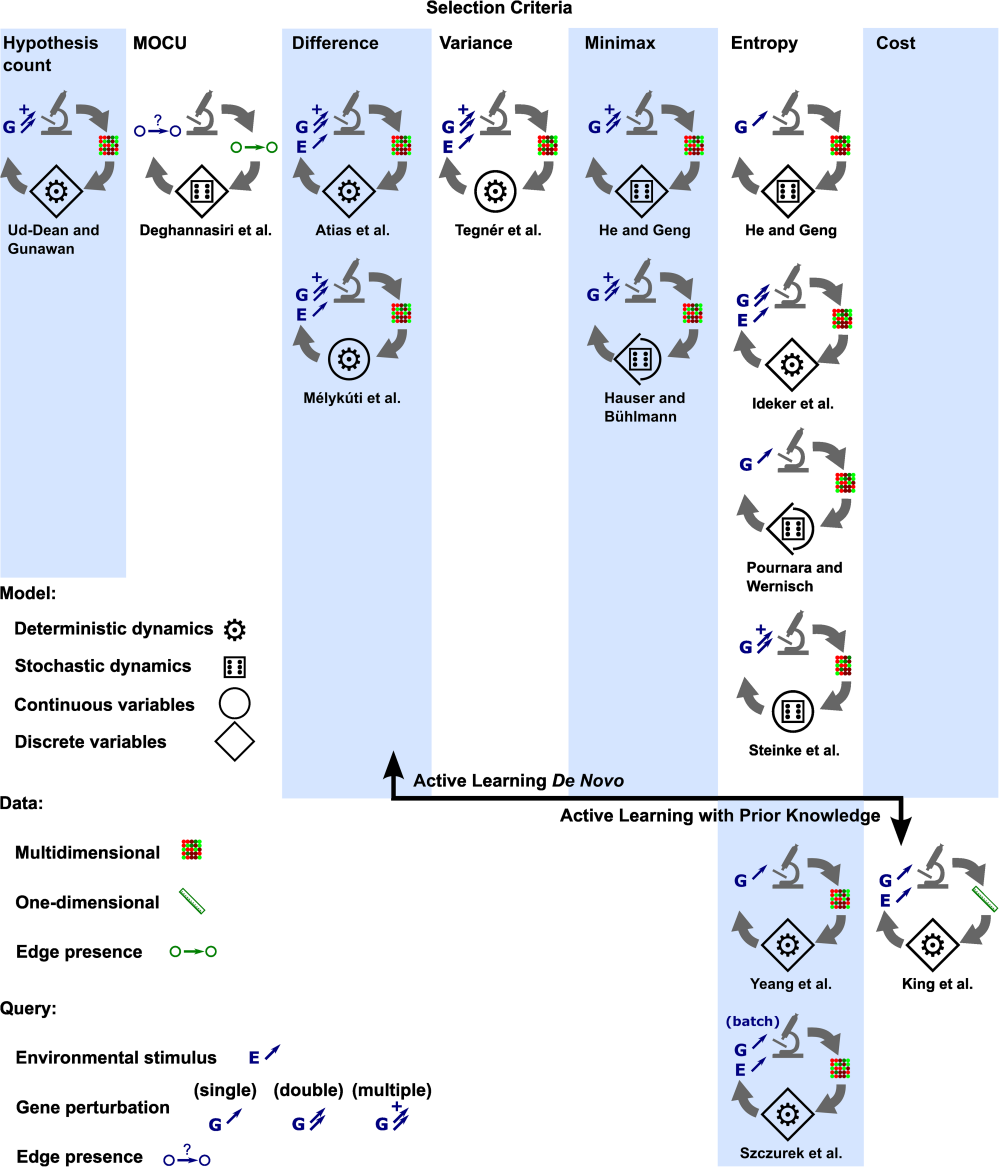
A brief summary of reviewed methods. Icons arranged in the table represent individual methods. The columns represent the various experiment selection criteria, and the methods are divided vertically between de novo methods and methods that use prior knowledge. Visual elements in each icon indicate whether the method is deterministic (cog) or stochastic (die), whether it models continuous (circle) or discrete (diamond) variables, what is specified in a query for an experiment (G for genetic and E for environmental perturbations), and the dimensionality of the data used (dot array for multidimensional data and a ruler for one-dimensional data).

There are several dimensions across which methods for active learning of biological networks can be readily compared. Fig 2 summarizes the models discussed in this review in terms of these dimensions. The defining feature of active learning is the selection of experiments to improve the underlying model. The majority of methods reviewed here use entropy or a related measure (such as information gain or Kullback–Leibler divergence) to select experiments. The intuitive explanation for the effectiveness of these measures is that the experiments that lead to high entropy in the model space or outcome space highly fragment those spaces. This means that a single outcome of the experiment will select one of many small fragments of the space, thereby significantly narrowing down that space.

Other experiment selection criteria also share themes across approaches. Both King et al. and Dehgahannasiri et al. minimize a variant of expected cost. Both Atias et al. and and Mélykúti et al. maximize the difference between predicted outcomes of competing models. Somewhat similarly, Tegnér et al. select experiments that maximize variation in predicted expression among perturbed genes across competing solutions. Minimax criteria are explored by He and Geng as well as Hauser and Bühlmann. Ud-Dean and Gunawan select background knockout combinations that can be used to test a maximal number of hypotheses about individual edges.

Fig 2 also characterizes multiple modeling aspects in the methods. The models by Ud-Dean and Gunawan and by Yeang et al. only represent characteristics of the edges in the network structure and treat those as discrete variables. The other approaches reviewed involve some modeling of gene expression levels or molecular abundances, which may be represented as discrete or continuous variables, where the values of the variables are viewed as deterministic quantities or as random quantities. The methods also differ in whether they have mechanisms for incorporating prior knowledge and how this knowledge is incorporated.

We find that the entropy-based criteria have the advantage of following a simple mathematical paradigm that translates well across models: there is typically a natural way to express entropy in a model space, whether the model is a Boolean network, a Bayesian network, a differential equation, a physical network, etc. The main weakness is that the entropy calculation requires counting models in the model space or having an expression for the probability distribution over models. Since the model space is often large, this makes entropy computationally intractable to compute in many cases, and approximate methods such as sampling the model space need to be used. The problem of computational complexity applies to all the methods discussed here, as none of the methods have been demonstrated to scale well to large datasets of thousands of nodes.

Prior knowledge can be incorporated into the model in a "soft" way, as in Yeang et al., or in a "hard" way, as in Szczurek et al. and King et al. In the soft approach of Yeang et al., the prior knowledge contributes to the inference of the model, but it can be outweighed by evidence from experimental data. For example, a protein–protein interaction may have strong prior knowledge support but not be included in the final model if there are no experiments that support it. In the hard approach of Szczurek et al. and King et al., on the other hand, the prior knowledge is used as a fixed part of the inference logic, and inference is performed only about those parts of the model that are not determined by prior knowledge. The model in Szczurek et al. uses a fixed model of signaling pathways to infer relationships downstream of them, and the model in King et al. uses a fixed model of metabolic reactions to infer which genes code for which enzymes. The decision regarding how prior knowledge should be incorporated boils down to the relevance of the knowledge to the experimental data and amount of certainty one has about the knowledge: Yeang et al. allowed the possibility of data outweighing prior knowledge because the prior knowledge about interactions in the network was known to be uncertain and because many interactions may exist that are not relevant to the experiments performed. Conversely, the metabolic pathways used by King et al. and the signaling pathways used by Szczurek et al. are well-studied, and there is much less uncertainty about their validity, justifying the hard incorporation of this kind of prior knowledge.

There are tradeoffs in modeling decisions that apply more generally, beyond active learning. In general, discrete variable modeling and deterministic dynamics yield simpler modeling, both computationally and conceptually, which in turn makes these models easier to implement and easier to interpret. The main weakness of discrete variable modeling is the danger of oversimplification; for example, if genes are considered to follow a binary on-or-off state model, one might miss nuances in gene expression data in which a gene's expression may decrease partially in response to a perturbation. The main weakness of deterministic modeling is that if inherent noise is present in the data from variations in instrument performance, biological variation, or uncontrolled-for variables, a deterministic model may not be able to account for this variation. Conversely, continuous variable modeling and stochastic dynamics address these weaknesses but do so at the cost of higher complexity, which may lead to models that are more difficult to interpret and harder to implement in practice.

The approaches reviewed also vary in the types of queries that specify each experiment and in the types of data that an experiment yields. The query specification is often constrained to a perturbation in only one or two elements in the network, such as single-gene knockout/overexpression [3, 5, 10, 11] or a single- or double-gene perturbation paired with a simple environmental perturbation [1, 2, 35]. The approaches that select these simpler queries typically select from a predefined set of possible experiments to perform. In contrast, the approaches that select arbitrarily complex perturbations [4, 6, 10, 11, 21, 25] or higher-order multiple-gene knockouts [26] form queries constructively, rather than selecting from a set of possible experiments. An even clearer trend is seen in the sort of data that experiments yield, wherein most of the methods rely on multidimensional data from each experiment, such as a gene expression profile or a profile of differential expression calls. Only the method by King et al. [35] views each experiment as yielding a one-dimensional binary outcome.

The approaches reviewed are diverse in the particular type of biological knowledge captured, the details of the mathematical models used, the degree of reliance on prior knowledge, robustness to noisy data, the type of data used, and the the type of query that specifies each experiment. Most of the approaches reviewed use entropy-based criteria for experiment selection. It is likely that future active learning approaches will continue to use entropy and similar criteria, based on previously demonstrated success as well as a strong conceptual motivation. The main challenge for future active learning approaches is that of scaling to big datasets, as high-dimensional screens have become the norm in systems biology. Another scaling problem to overcome is that of proposing not single experiments but batches of experiments, something that is explicitly explored by Szczurek et al. in the context of discovering regulatory relationships downstream of a given signaling pathway. The method by Ud-Dean and Gunawan also explicitly considers batches of experiments but relies on the ability to determine the presence of individual interactions in a targeted way. There is still a need for methods that design batches of experiments outside of these specific settings. The ability to propose batches of experiments is important for real-world application because it is often more practical to perform multiple perturbation experiments simultaneously in the lab. The nature of experimentation is such that noisy observations are a reality due to uncontrollable factors and measurement errors. It is therefore important to develop methods that are robust to noise in observations, a challenge better addressed by models with stochastic dynamics. Finally, existing work demonstrates that the leveraging of prior knowledge has been essential in fruitful discovery of biological knowledge using active learning.
